# Supported Silver Nanoparticles as Catalysts for Liquid-Phase Betulin Oxidation

**DOI:** 10.3390/nano11020469

**Published:** 2021-02-12

**Authors:** Anna Grigoreva, Ekaterina Kolobova, Ekaterina Pakrieva, Päivi Mäki-Arvela, Sónia A. C. Carabineiro, Alina Gorbunova, Nina Bogdanchikova, Dmitry Yu. Murzin, Alexey Pestryakov

**Affiliations:** 1 Research School of Chemistry & Applied Biomedical Sciences, National Research Tomsk Polytechnic University, 634050 Tomsk, Russia; bar0710@mail.ru (A.G.); epakrieva@mail.ru (E.P.); aag84@tpu.ru (A.G.); 2 Johan Gadolin Process Chemistry Centre, Åbo Akademi University, 20500 Turku/Åbo, Finland; pmakiarv@abo.fi (P.M.-A.); dmurzin@abo.fi (D.Y.M.); 3 Centro de Química Estrutural, Instituto Superior Técnico, Universidade de Lisboa, 1049-001 Lisboa, Portugal; sonia.carabineiro@fct.unl.pt; 4 LAQV-REQUIMTE, Department of Chemistry, NOVA School of Science and Technology, Universidade NOVA de Lisboa, 2829-516 Caparica, Portugal; 5 Centro de Nanociencias y Nanotecnología, Universidad Nacional Autónoma de México, Ensenada 22800, Mexico; nina@cnyn.unam.mx

**Keywords:** silver catalyst, betulin oxidation, alumina polymorphic modification, acid-base properties of support, structure sensitivity

## Abstract

Herein, it has been shown that betulin can be transformed into its biologically active oxo-derivatives (betulone, betulinic and betulonic aldehydes) by liquid-phase oxidation over supported silver catalysts under mild conditions. In order to identify the main factors determining the catalytic behavior of nanosilver catalysts in betulin oxidation, silver was deposited on various alumina supports (γ-alumina and boehmite) using deposition–precipitation with NaOH and incipient wetness impregnation methods, followed by treatment in H_2_ or O_2_. Silver catalysts and the corresponding supports were characterized by X-ray diffraction, nitrogen physisorption, inductively coupled plasma optical emission spectroscopy, photoelectron spectroscopy and transmission electron microscopy. It was found that the support nature, preparation and treatment methods predetermine not only the average Ag nanoparticles size and their distribution, but also the selectivity of betulin oxidation, and thereby, the catalytic behavior of Ag catalysts. In fact, the support nature had the most considerable effect. Betulin conversion, depending on the support, increased in the following order: Ag/boehmite < Ag/boehmite (calcined) < Ag/γ-alumina. However, in the same order, the share of side reactions catalyzed by strong Lewis acid centers of the support also increased. Poisoning of the latter by NaOH during catalysts preparation can reduce side reactions. Additionally, it was revealed that the betulin oxidation catalyzed by nanosilver catalysts is a structure-sensitive reaction.

## 1. Introduction

Betulin (CAS 473-98-3 betulenol C_30_H_50_O_2_) is a pentacyclic triterpene alcohol of the lupane series with a broad spectrum of pharmacological activity. In the composition of plant membranes, the presence of betulin is necessary to protect the plant from damaging environmental factors such as radiation, bacteria, fungi, viruses, and insects. While several plants contain betulin in small amounts, the main source of this compound is the outer part of the birch bark, with the contents varying from 10% to 35%. The amount of betulin depends on the age of the birch tree, its location and type and some other factors [1]. It should also be noted that birch bark is a large-tonnage waste from the wood processing industry. At the same time, betulin can be relatively easily isolated by extraction of the birch bark with various solvents using infusion, reflux method, alkaline hydrolysis of birch bark followed by extraction of betulin, “explosive” autohydrolysis, etc. [1,2,3,4,5,6].

The interest in the study of betulin and its oxo-derivatives has grown significantly over the last twenty years. A seminal study of Pisha et al. appeared in 1995 [7], positioning betulinic acid as a potential selective inhibitor of human melanoma cells. Lately, activity against other types of cancer, such as lung carcinoma [8], hepatic [9] and breast cancer [10] was demonstrated, along with anti-inflammatory [11,12,13], antimicrobial [14,15], anti-fibrotic [16], antiviral [17] and other properties.

Betulonic acid, another valuable derivative, also has a wide spectrum of biological activity. Strong cytotoxic [18], anti-leishmanial [19], anti-inflammatory [20] and antimicrobial [21] properties were found. Betulinic and betulonic aldehydes demonstrated mainly antitumor activity. In particular, betulinic aldehyde shows an inhibitory effect on leukemia, melanoma, and the growth of neuroblastoma cells [22]. Betulonic aldehyde has exhibited strong cytotoxicity against lung adenocarcinoma cells, small carcinoma cells, and giant carcinoma cells [23]. Another oxo-derivative of betulin is betulone, which has an antitumor activity and is also a key building block for the synthesis of a number of compounds [18,24,25].

It should be noted that betulin is capable of affecting metabolism at the cellular level, namely respiration and oxidative phosphorylation of rat liver mitochondria, but only in high concentrations (50 μM) [26]. Moderate concentrations of betulin in the range of 11–20 µM [27,28] demonstrate an ability to selectively suppress tumor growth without affecting the growth of normal cells. Betulin does not have a toxic effect at high doses: once intragastrically at a dose of 1000–16,000 mg/kg and once intraperitoneally at a dose of 250–4000 mg/kg in mice and rats [29]. After injections, the authors observed the animals for 14 days. They did not reveal, taking into account the data of necropsy, any pathological changes and toxic effects of betulin. Additionally, according to [30], betulin is a non-toxic compound and can be classified as a substance of VI class toxicity.

The main method for the preparation of oxo-derivatives of betulin is its oxidation with toxic compounds: chromium (the Jones reagent [31], complexes with pyridinium chromates [32], etc.), manganese (two-stage Swern-type oxidation of betulin and then with a solution of KMnO_4_ in a mixture 1,4-dioxane in water [33]), as well as mixtures of 2,2,6,6-Tetramethylpiperidin-1-yl)oxyl (TEMPO) with NaClO_2_-NaOCl [34]. Moreover, the two-stage Swern-type oxidation of betulin, followed by the oxidizing system NaClO_2_, H_2_O_2_ and NaH_2_PO_4_·H_2_O in *t*-BuOH in water was reported [35]. Since these methods do not correspond to the principles of green chemistry, numerous efforts were focused on finding alternative ways to obtain oxo-derivatives of betulin.

There are few studies on the preparation of oxo-derivatives of betulin via genetic engineering. Thus, betulinic acid was obtained in *Saccharomyces cerevisiae* from endogenous yeast 2,3-oxidosqualene by successfully combining the expression of the C_28_ oxidase from C. roseus and lupeol synthase from *Arabidopsis thaliana* [36]. 

Several reports were devoted to formation of betulin derivatives using microorganisms [37]. For this purpose, various filamentous fungi were screened for the biotransformation of betulin to betulinic acid. Among the fungi tested, two strains of *Aspergillus* (*A. foetidus ZU-G1* and *A. Oryzae AS 3.498*) demonstrated a significant ability to oxidize the C-28 hydroxyl group to the corresponding carboxylic acid [38]. Feng et al. obtained betulin derivatives by microbial transformation with *Cunninghamella blakesleeana* [39]. Marine mushroom Dothideomycetesp. HQ 316564 [40] and the yeast strain *Rhodotorula mucilaginosa* [41] catalyzed the oxidation of betulin to betulone. It should be noted that betulone is obtained predominantly by a microbiological method, because this procedure avoids tedious stages of protection/deprotection of functional groups, which should not be oxidized [41].

Several studies have been devoted to the preparation of oxo-derivatives of betulin using homogeneous catalysts RuCl_2_(PPh_3_)_3_ and Pd(OAc)_2_ mixed with TEMPO, triethylamine or pyridine [42,43]. However, these catalytic systems turned out to be ineffective. The maximum betulin conversion achieved using these systems amounted 17% and 42% [43], respectively. Immobilization of Pd(OAc)_2_ on the hydrotalcite surface afforded 99% conversion of betulin with selectivity for betulinic and betulonic aldehydes of 21% and 28%, respectively, and unfortunately 51% of unidentified reaction products [43].

Previously, some of the authors of the current work [44,45,46,47,48], demonstrated that oxo-derivatives of betulin can be obtained by oxidation with synthetic air under mild conditions using heterogeneous catalysts based on Pd, Ru, Au, and Ag nanoparticles. In the case of Pd-containing catalysts [48], the maximum conversion of betulin was 10%. Catalytic systems based on Ru nanoparticles supported on various carbon supports (active carbon, carbon nanotubes, graphitic carbon nitride, N-doped carbon, carbon nanofibers and a mesoporous carbon support Sibunit) turned out to be more efficient for betulin oxidation in comparison with Pd catalysts [47,48]. The best results were obtained with Ru/C as a catalyst mixed with basic hydrotalcite and SiO_2_ as a dehydrating agent at 108 °C in toluene with air as an oxidizing agent. Under these conditions, the betulin conversion after 24 h was 41% with a betulinic aldehyde selectivity of 67%. It should be noted that higher conversions (54% and 70%) were obtained using Ru/C (without hydrotalcite and SiO_2_) and Ru/carbon nitride, respectively. However, in the first case, the main reaction product was allobetulin. In the case of Ru/carbon nitride, the total selectivity for the main products (betulone, betulinic and betulonic aldehydes, betulinic and betulonic acids) did not exceed 47%. Catalytic systems based on gold nanoparticles supported on various oxides (unmodified and modified titania, hydrotalcite, magnesia, ceria, lanthana, zirconia, zinc oxide, alumina and boehmite) were investigated as catalysts for the liquid-phase oxidation of betulin [44,45]. Up to now, the best results among all heterogeneous catalysts for the liquid-phase oxidation of betulin studied by the authors were obtained on a gold-containing catalyst with boehmite (Au/AlOOH_C) as a support [45]. Conversion of betulin on this catalyst was 77% with selectivity for betulone 47%, betulonic aldehyde 34%, and betulinic acid 17% at 140 °C, using mesitylene as a solvent.

Silver-containing systems have also been studied as catalysts for the liquid-phase oxidation of betulin. The best results were obtained using Ag/CeO_2_/TiO_2_ with a betulin conversion of 27% in 6 h. The main reaction product was betulone with a selectivity of 60% [46]. Although conversion was relatively low, this study was one of the few reports [49] devoted to the study of catalysts based on silver nanoparticles in the liquid-phase oxidation of alcohols. The results have demonstrated the potential of silver catalysts with a nanosized active phase for such reactions because their activity can be significantly improved by optimizing the composition, as well as pretreatment conditions and preparation methods.

This work is aimed at further investigating of silver-containing catalysts for liquid-phase oxidation of betulin with synthetic air, revealing the effect of the preparation method, the support nature and pretreatment atmosphere on their catalytic behavior.

## 2. Materials and Methods

γ-Al_2_O_3_ (Versal gamma alumina (VGL-25), UOP, Des Plaines, IL, USA) and AlOOH (boehmite (Catapal B), Sasol, Hamburg, Germany), as well as the material designated as AlOOH_cal obtained by calcining AlOOH (boehmite (Catapal B), Sasol, Hamburg, Germany) at 600 °C for 3 h, were used as supports.

Silver catalysts (denoted as Ag/γ-Al_2_O_3__dp, Ag/AlOOH_dp and Ag/AlOOH_cal_dp) were prepared by deposition–precipitation with NaOH (DP with NaOH), according to the procedure previously described [46]. To an aqueous solution containing the calculated amount of AgNO_3_ (Merck, Darmstadt, Germany), 1 g of the support (γ-Al_2_O_3_, AlOOH or AlOOH_cal) was added followed by heating to 80 °C. The pH, being initially ca. 3, was adjusted to 9 by dropwise addition of a 0.5 M NaOH solution. The suspension was kept at 80 °C for 2 h under vigorous stirring. After deposition, all samples were washed and centrifuged several times, and then dried in vacuum for 2 h at 80 °C.

Silver catalysts (denoted as Ag/γ-Al_2_O_3__iw, Ag/AlOOH_iw and Ag/AlOOH_cal_iw) were prepared by incipient wetness impregnation (IWI) of alumina supports with an aqueous solution containing the calculated amounts of AgNO_3_ (Merck, Darmstadt, Germany), according to the procedure previously described [50,51]. Then, the obtained materials were dried at 150 °C for 3 h.

The nominal silver content was 3 wt.% in all catalysts. After the basic procedures of silver deposition and drying, all the obtained materials were pretreated either in H_2_ or O_2_.

The obtained catalysts are denoted hereinafter as Ag/γ-Al_2_O_3__dp(iw)_P, Ag/AlOOH_dp(iw)_P and Ag/AlOOH_cal_dp(iw)_P, where dp or iw indicate the used catalyst preparation method (deposition–precipitation with NaOH or incipient wetness impregnation) and P indicates the pretreatment atmosphere (H_2_ or O_2_).

X-ray diffraction (XRD) was performed by the step-scanning procedure on a MiniFlex II diffractometer (RIGAKU, Tokyo, Japan), using CuKα radiation. The conditions were: scanning speed 1 deg/min, radiation intensity 1000 units, tube voltage 30 kV, current 15 mA. The phase composition of catalysts and the corresponding supports were determined by Crystallographica Search-Match Version 2.1.1.1. reference databases.

The textural properties of catalysts and corresponding supports were studied using an ASAP 2060 (Micromeritics Instrument Corporation, Norcross, GA, USA) apparatus. Prior to measurements, the materials were degassed in vacuum at 300 °C for 5 h. The Brunauer-Emmett-Teller (BET) method was used to calculate the specific surface area from the adsorption data (in the range of relative pressures from 0.005 to 0.25). The pore size distribution was calculated from the desorption data using the Barrett-Joyner-Halenda (BJH) method.

The Ag loading of the catalysts was measured by inductively coupled plasma optical emission spectrometry (ICP-OES) using a Horiba Jobin Yvon (Longjumeau, France) Ultima apparatus. The solids were treated by aqua regia, digested in the microwave oven, diluted to 100 mL and analyzed in the spectrometer.

The chemical state of silver was determined by XPS using a VG Scientific ESCALAB 200A spectrometer (Thermo Fisher Scientific, Waltham, MA, USA) with Al Kα radiation (1486.6 eV) at the Centro de Materiais da Universidade do Porto (CEMUP). The charge effect was corrected using the C1s peak as a reference (binding energy of 285 eV). The CASA XPS software (version 2.3.15, CASA Software Ltd., Teignmouth, UK, http://www.casaxps.com/ (accessed on 10 February 2021)) was used for data analysis.

The size of silver nanoparticles and their distribution were studied by high resolution transmission electron microscopy (HRTEM) using a JEM-2100F microscope (JEOL Ltd., Tokyo, Japan). The samples were ground to a fine powder and sonicated in hexane at room temperature. Then, a part of the suspension was placed on a lacey carbon-coated Cu grid. To obtain images that most fully reflected the real structure of the samples, a general survey of the samples was carried out, thereafter the selected area was scanned at higher resolutions. For each sample, at least 500 particles were counted. The ImageJ program (https://imagej.net/ (accessed on 10 February 2021)) was used to measure the particle size.

Betulin (94–99%) was purchased from Betulika Co., Ltd., (Yekaterinburg, Russia). The procedure for betulin oxidation and the analytical methods for determining changes in the concentration of reagents and products during the reaction, are described in detail in our previous works [44,45,46,47,48]. Briefly, the catalytic behavior of various Ag/alumina materials was studied in the liquid-phase betulin oxidation with synthetic air at 140 °C, using mesitylene as a solvent. The initial concentration of betulin in mesitylene and the catalyst loading were 4.52 mmol/L and 0.2 g, respectively. To confirm the reliability of the results of catalytic studies, catalytic tests were performed twice for one catalyst from each series of catalysts prepared by the same preparation method, namely for Ag/γ-Al_2_O_3__dp_pH_2_, Ag/γ-Al_2_O_3__iw_pH_2_, Ag/AlOOH_dp_pH_2_, Ag/AlOOH_iw_pH_2_, Ag/AlOOH_cal_dp_pH_2_ and Ag/AlOOH_cal_iw_pH_2_.

To monitor the progress of the reaction, the aliquots were withdrawn from the reactor at regular intervals and analyzed by GC (PerkinElmer AutosystemXL, Waltham, MA, USA) equipped with an Agilent HP-1 (Santa-Clara, CA, USA) capillary column. Prior to GC-analyses, the aliquots were silylated by a mixture of pyridine, N,O-bis(trimethylsilyl)trifluoroacetamide and trimethylsilyl chloride in a 1:4:1 volume ratio followed heating in an oven at 70 °C for 45 min. To mitigate potential large errors, GC injections for each measurement were made twice and the average error dispersion was lower than 5%.

Turnover frequency (TOF) values were calculated according to the following equation:

(1) $$TOF=\;\frac{kC_{0}V}{60n_{Ag}D}$$

where *C*_0_ is the initial betulin concentration (mol/L), *V* is volume of reaction media (L), *k* is first order reaction rate constant (s^−1^), *n_Ag_* is the number of moles of Ag and *D* is dispersion. The reaction rate constant (*k*) was determined by numerical data fitting of the concentration curves using the first order kinetics. The number of surface metal atoms was calculated knowing the average silver particle size measured by transmission electron microscopy (TEM), according to the following equation:

(2) $$D=\;\frac{6M}{\rho d_{Ag}N_{A}cs}$$

where *d_Ag_* is the average silver particle size, *ρ* and *cs* are its bulk density and cross-section, *N_A_* is the Avogadro number.

## 3. Results and Discussion

### 3.1. Characterization Results

X-ray diffraction (XRD) was used to investigate the phase composition of the studied Ag catalysts and the corresponding supports. For a series of Ag catalysts based on γ-Al_2_O_3_ and AlOOH supports, Figure S1 and Table 1 present only the data for H_2_-treated samples, while, for the Ag/AlOOH_cal series of materials, the data correspond to the samples after treatment in H_2_ and O_2_. Analysis of X-ray powder diffraction data of supports showed that two of the three used supports (designated as γ-Al_2_O_3_ and AlOOH_cal) had a diffraction pattern typical of γ-alumina with a cubic crystal lattice. The material designated as AlOOH is boehmite with an orthorhombic crystal lattice (Table 1).

After silver deposition on the supports surface, regardless of the method of preparation and pretreatment, the structure and composition of the supports did not change, except Ag/AlOOH_cal_dp_pO_2_, which in addition to the main γ-Al_2_O_3_ phase had reflections characteristic for bayerite (Figure S1, Table 1). It is known that γ-Al_2_O_3_ is unstable under hydrothermal conditions [52,53,54,55,56]. The surface of the particles is hydrated to form amorphous aluminum hydroxide, aluminum trihydroxides and boehmite depending on the conditions. Exposure at 25–55 °C in a humid environment and atmospheric pressure is accompanied by the formation of amorphous aluminum hydroxide on the surface of the particles, which then crystallizes into bayerite. For example, the authors [56] observed the simultaneous formation of gibbsite and bayerite. Their fraction reached 18 wt.% after 150 h in an aqueous suspension at pH = 10. Previously, formation of the boehmite phase (7 wt.%) after gold deposition on the γ-Al_2_O_3_ surface was reported for deposition–precipitation with urea (pH = 9) [45]. In the present study, Ag/AlOOH_cal_dp_pO_2_ was prepared by DP with NaOH followed by oxidative pretreatment at 300 °C. The XRD pattern of as-prepared Ag/AlOOH_cal also contains reflexes characterizing the presence of the bayerite phase (Figure S1). It is worth noting that the bayerite phase disappeared when the oxidative treatment was replaced by a reductive one (Figure S1, Table 1, Ag/AlOOH_cal_dp_pH_2_). 

The metallic silver phase was detected by XRD for all Ag/alumina materials. However, the intensity of the reflections characterizing the silver phase is very low and the corresponding reflections are practically invisible against the background of the supports. In the diffraction pattern, only a slight broadening of the diffraction peaks and an insignificant shift of the main reflections of γ-Al_2_O_3_ or AlOOH are noticeable compared to the materials without silver. These data indicate the absence of large metallic Ag particles in the prepared materials or their X-ray amorphous structure. It should be noted that characteristic reflections of the Ag_2_O phase were not found in the XRD pattern of studied catalysts, indicating the decomposition of silver precursors (AgNO_3_ and Ag_2_O) with the formation of Ag metal nanoparticles upon exposure to both oxidative and reductive treatment. In the previous studies, it was shown by temperature-programed reduction that the silver precursor (Ag_2_O) on M_x_O_y_/TiO_2_ (M_x_O_y_–Fe_2_O_3_, CeO_2_ or MgO) decomposes under reductive treatment at 50–120 °C (AgNO_3_) [46] and decomposes on the zeolite surface at 120–220 °C [50].

Table 1 also shows the textural characterization (BET surface area, pore size and pore volume), the average size of silver nanoparticles and the silver content. The latter, determined using ICP, varies in the range of 2.6–2.8 wt.%, being close to its nominal content of 3 wt.%.

According to the low-temperature adsorption-desorption data of N_2_ (Table 1), the supports in this work are characterized by a high specific surface area and a mesoporous structure. At the same time, boehmite has the largest specific surface area, but the smallest pore size and volume among the tested supports. Calcination of boehmite at 600 °C for 3 h leads to the formation of a γ-alumina (AlOOH_cal) phase, which is accompanied by a decrease in the specific surface area and an increase in the size and volume of pores. After silver deposition, a significant decrease in the specific surface area with a simultaneous increase in the pore size and volume in comparison with the corresponding supports was observed only for boehmite. As was discussed in the previous work [45], such changes probably originate from an increase in the average crystal size of the supports after the metal deposition, which may be due to the partial dehydroxylation of the support surface under the action of high-temperature treatment at 300 °C, as well as a consequence of the interaction of the metal precursor with the support surface during catalyst preparation. An increase in the crystal lattice parameters when boehmite is heated from 195 °C to 257 °C was previously reported [57,58,59]. Above 257 °C, the γ-Al_2_O_3_ phase starts to be formed during the thermal decomposition of boehmite.

**Figure 1:**
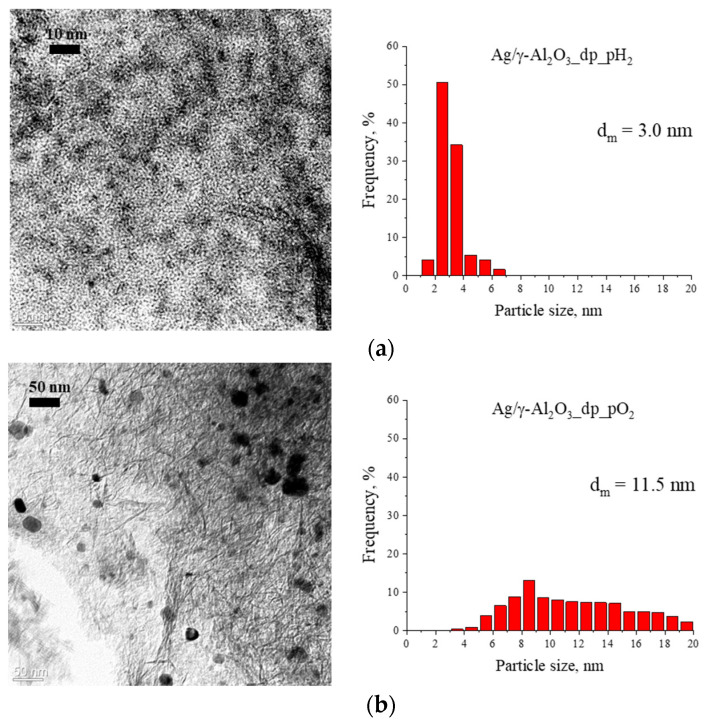

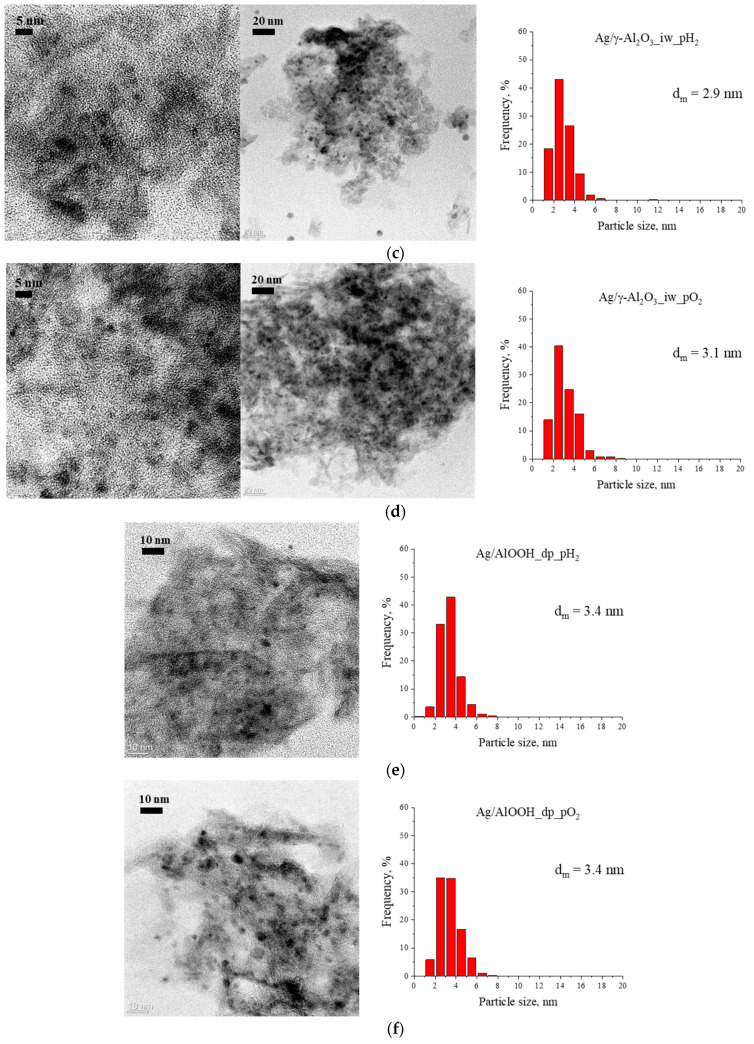

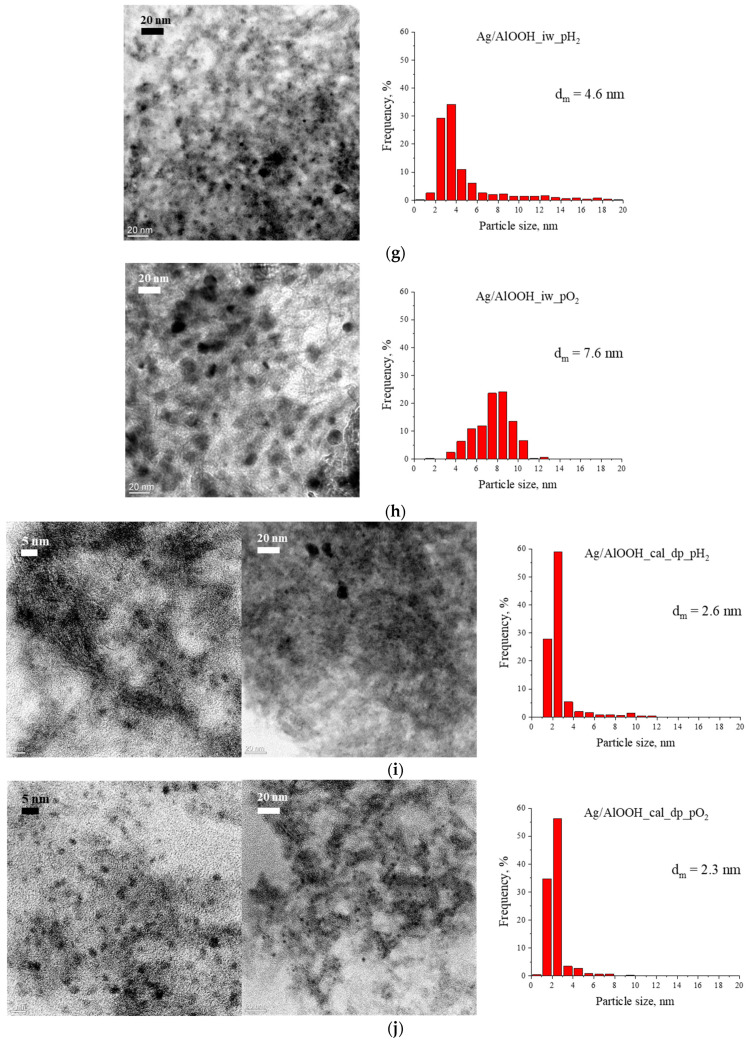

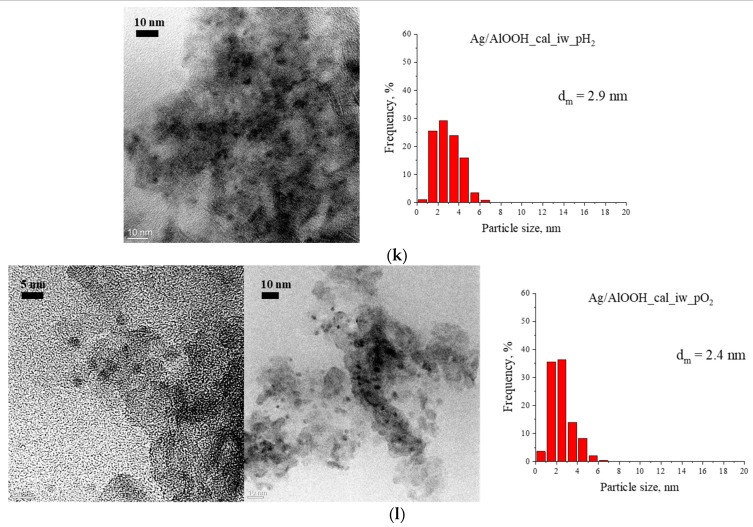
TEM micrographs and Ag particle size distribution of the studied catalysts: (**a**) Ag/γ-Al_2_O_3__dp_pH_2_; (**b**) Ag/γ-Al_2_O_3__dp_pO_2_; (**c**) Ag/γ-Al_2_O_3__iw_pH_2_; (**d**) Ag/γ-Al_2_O_3__iw_pO_2_; (**e**) Ag/AlOOH_dp_pH_2_; (**f**) Ag/AlOOH_dp_pO_2_; (**g**) Ag/AlOOH_iw_pH_2_; (**h**) Ag/AlOOH_iw_pO_2_; (**i**) Ag/AlOOH_cal_dp_pH_2_; (**j**) Ag/AlOOH_cal_dp_pO_2_; (**k**) Ag/AlOOH_cal_iw_pH_2_; (**l**) Ag/AlOOH_cal_iw_pO_2_.

Figure 1 shows the size distribution of silver particles and TEM images for Ag catalysts. For the majority of the studied materials, the distribution interval of Ag nanoparticles (NPs) varies from 1 to 9 nm with an average particle size between 2.3 to 3.4 nm. For Ag/AlOOH_cal_iw_pH_2_, Ag/AlOOH_cal_iw_pO_2_ and Ag/AlOOH_cal_dp_pO_2_ silver particles with a size below 1 nm (up to 3% of the total number of particles) were also found (Figure 1j–l). For Ag/AlOOH_cal_dp_pH_2_ the average Ag size was 2.6 nm. For this catalyst, a broad distribution of Ag particles (1–12 nm) was observed, with a large fraction of particles (85%) being 1–3 nm in size, while the fraction of large particles (>5 nm) was less than 5% (Figure 1i). Larger Ag particles (4.6–11.5 nm) distributed over a wide range (1–20 nm) were found on the surface of Ag/AlOOH_iw_pH_2_, Ag/AlOOH_iw_pO_2_ and Ag/γ-Al_2_O_3__dp_pO_2_ (Figure 1b,g,h). It was concluded in [60,61] that the size and distribution of silver nanoparticles very strongly depends on the polymorphic modification of alumina, as well as the size of the alumina crystals [62]. Formation of larger particles on the surface of γ-Al_2_O_3_ was attributed [60] to weaker interactions of the metal with the support exhibiting a lower content of OH-groups, compared to γ-Al_2_O_3_ obtained from AlOOH. For the amorphous structure of the support obtained from Al(OH)_3_, the particle size was the largest one due to the formation of larger particles of silver oxide Ag_2_O and, subsequently, silver particles. In the present study, a similar analogy can be drawn between the nature of alumina support and the average size of Ag NPs. Silver particles of the smallest size (2.3–2.9 nm) were formed on the surface of AlOOH_cal, obtained by calcining boehmite at 600 °C for 3 h. Furthermore, there were materials which had γ-Al_2_O_3_ as a support with the average size of silver of ca. 3 nm, except Ag/γ-Al_2_O_3__dp_pO_2_, showing the largest particles (11.5 nm) among the silver supported on alumina (Figure 1b). Ag particles larger than those of AlOOH_cal and γ-Al_2_O_3_ were found on the boehmite surface (3.4–7.6 nm).

It should also be noted that for all catalysts, without any exception, smaller particles were formed using DP with NaOH, which is probably due to the initial deposition of Ag_2_O, rather than AgNO_3_, which was the case of IWI method. The exception was Ag/γ-Al_2_O_3__dp_pO_2_ (Figure 1b). In addition, for six of the twelve studied materials representing Ag/alumina (Ag/γ-Al_2_O_3__dp_pH_2_ and Ag/γ-Al_2_O_3__dp_pO_2_, Ag/γ-Al_2_O_3__iw_pH_2_ and Ag/γ-Al_2_O_3__iw_pO_2_, Ag/AlOOH_iw_pH_2_ and Ag/AlOOH_iw_pO_2_, Figure 1a–d,g,h) with the same preparation method and the support, larger Ag particles were formed after the oxidative treatment. Giorgio et al. [63,64] demonstrated a change in the shape of particles under the influence of H_2_ or O_2_. Particles in the form of a truncated octahedron were formed when the hydrogen treatment was applied, while exposure to O_2_ resulted in rounding and de-wetting of nanoparticles. At the same time, for AlOOH_cal (Figure 1i–l)-based materials an inverse dependence of the average size on the nature of pretreatment (H_2_ or O_2_) was seen, namely smaller particles were found on the surface of O_2_-treated catalysts. A likely explanation is the different nature of interactions between silver particles of different sizes with oxygen. Bukhtiyarov et al. [65] observed sintering of Ag NPs due to oxygen treatment and, as a consequence, an increase in the size of nanoparticles if the average size is in the range of 2–4 nm. At the same time, catalysts with an average size of Ag NPs in the range of 1–2 nm occurred under the action of oxygen oxidation of metal particles with the formation of finely dispersed Ag_2_O.

The nature of the pretreatment (H_2_ or O_2_) did not significantly affect the size and distribution of silver nanoparticles on the surfaces of Ag/AlOOH_dp_pH_2_ and Ag/AlOOH_dp_pO_2_ (Figure 1e,f). The average size of silver particles for both catalysts was 3.4 nm. It should be mentioned separately that for almost all studied materials, a large fraction of silver particles has a spherical/hemispherical shape, except Ag/γ-Al_2_O_3__iw_pH_2_ (Figure 1c), where a large number of elliptical particles along with the spherical/hemispherical particles were also found.

**Figure 2:**
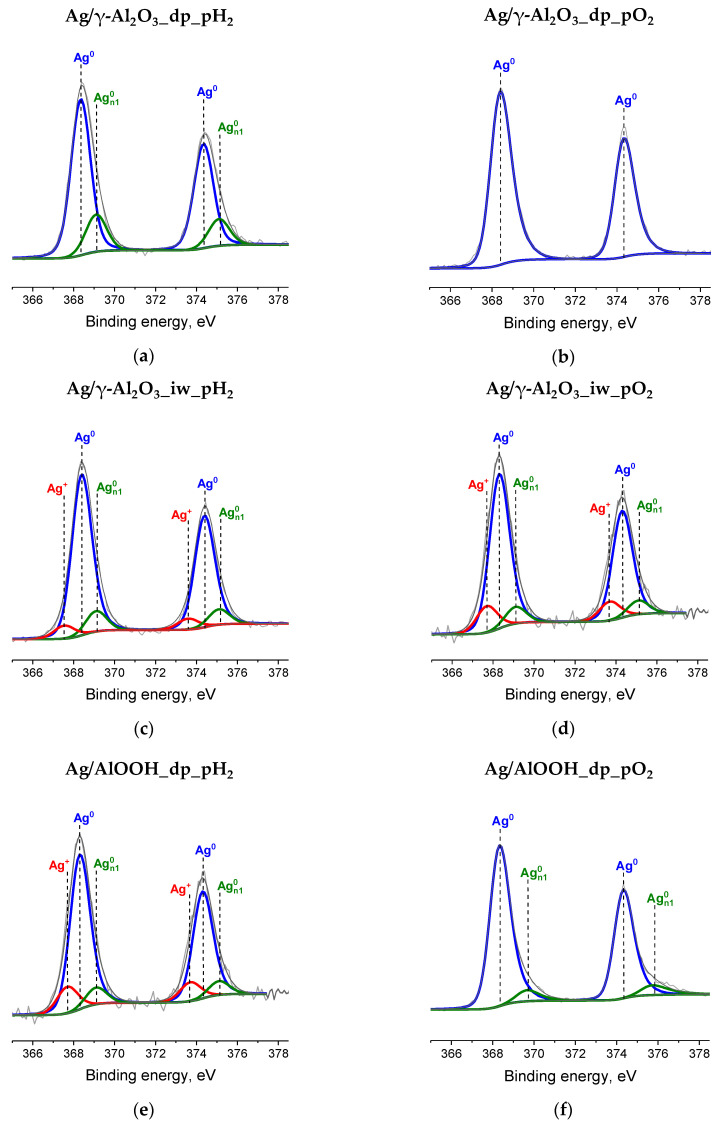

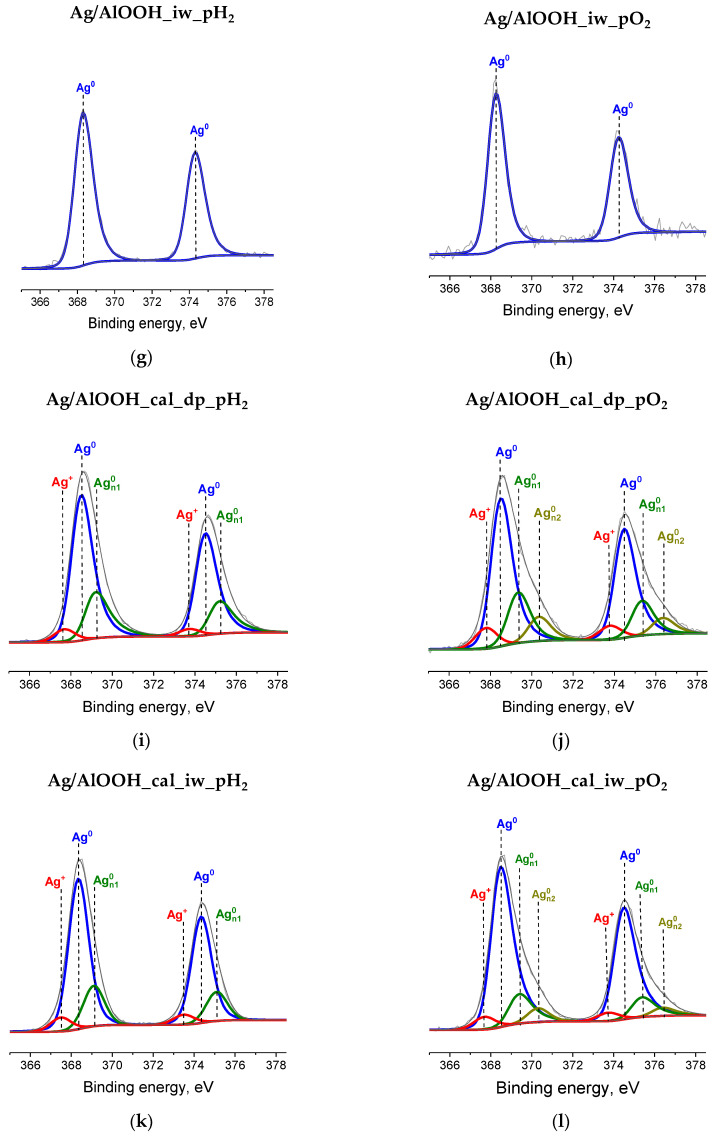
Ag 3d XPS spectra of studied catalysts: (**a**) Ag/γ-Al_2_O_3__dp_pH_2_; (**b**) Ag/γ-Al_2_O_3__dp_pO_2_; (**c**) Ag/γ-Al_2_O_3__iw_pH_2_; (**d**) Ag/γ-Al_2_O_3__iw_pO_2_; (**e**) Ag/AlOOH_dp_pH_2_; (**f**) Ag/AlOOH_dp_pO_2_; (**g**) Ag/AlOOH_iw_pH_2_; (**h**) Ag/AlOOH_iw_pO_2_; (**i**) Ag/AlOOH_cal_dp_pH_2_; (**j**) Ag/AlOOH_cal_dp_pO_2_; (**k**) Ag/AlOOH_cal_iw_pH_2_; (**l**) Ag/AlOOH_cal_iw_pO_2_.

XPS was used to study the electronic state of silver on the surface of Ag/alumina materials (Figure 2, Table 2). Table 2 summarizes the XPS data—the chemical state of silver on the surface (Ag^+^, Ag^0^, ${\mathrm{Ag}}_{n1}^{0}$ and ${\mathrm{Ag}}_{n2}^{0}$)—and their relative atomic concentrations obtained by calculating the area of the corresponding deconvoluted peaks.

Most of silver (69–100%) present in the studied catalysts was in the metallic state with binding energy (BE) (Ag3d_5/2_) 368.2–368.3 eV [66,67]. Moreover silver was found on the surface of Ag/γ-Al_2_O_3__dp_pO_2_, Ag/AlOOH_dp_pH_2_, Ag/AlOOH_iw_pH_2_, Ag/AlOOH_iw_pO_2_ only in the metallic state (Figure 2b,e,g,h). The silver content in the ionic state (Ag^+^) with BE (Ag3d_5/2_) in the range 367.5–367.7 eV [68,69,70] was 5–8% for materials with an average size of Ag NPs lower than 3 nm and/or pretreated in O_2_, in particular, Ag/γ-Al_2_O_3__iw_pH_2_ (2.9 nm, Figure 2c) and Ag/γ-Al_2_O_3__iw_pO_2_ (3.1 nm, Figure 2d) and a series of materials with AlOOH_cal as a support (Ag NPs less than 3 nm, Figure 2j–l).

The state of silver with BE (Ag3d_5/2_) 369.1–369.3 eV and 370.2 eV refers to highly dispersed metal particles/clusters lower than 2 nm. A positive shift of BE by 1–2 eV in XPS spectra relative to the standard value BE (Ag3d_5/2_) = 368.2 eV for the metallic state can be observed in the presence of such particles [71,72,73]. It is worth noting that two states of silver with BE (Ag3d_5/2_) 369.2 eV and 370.2 eV were simultaneously detected by XPS only for Ag/AlOOH_cal_dp_pO_2_ and Ag/AlOOH_cal_iw_pO_2_ (Figure 2j,l). These states correspond to highly dispersed metallic particles/clusters, which may be associated with fine particles/clusters of different sizes.

The XPS results are in good agreement with the TEM data. In particular, in the XPS spectra of Ag/AlOOH_cal_dp_pO_2_ and Ag/AlOOH_cal_iw_pO_2_ (Figure 2j,l), two states of silver were found related to particles/clusters lower than 2 nm. The same materials were characterized by the presence of Ag NPs of the smallest size (2.3–2.4 nm, Figure 1j,l) among all studied catalysts. In the XPS spectra of catalysts containing Ag NPs with an average size higher than 3 nm (3.4–11.5 nm), silver was found only in the metallic state. An exception is Ag/AlOOH_dp_pO_2_, which had the ground state with BE (Ag3d_5/2_) = 368.3 eV, corresponding to Ag^0^, and the state (${\mathrm{Ag}}_{n1}^{0}$), related to fine metal particles/clusters (Figure 2f). At the same time, according to TEM data, the fraction of particles with a size in the range of 1–2 nm for this material was 6% (Figure 1f).

### 3.2. Catalytic Results

**Figure 3:**
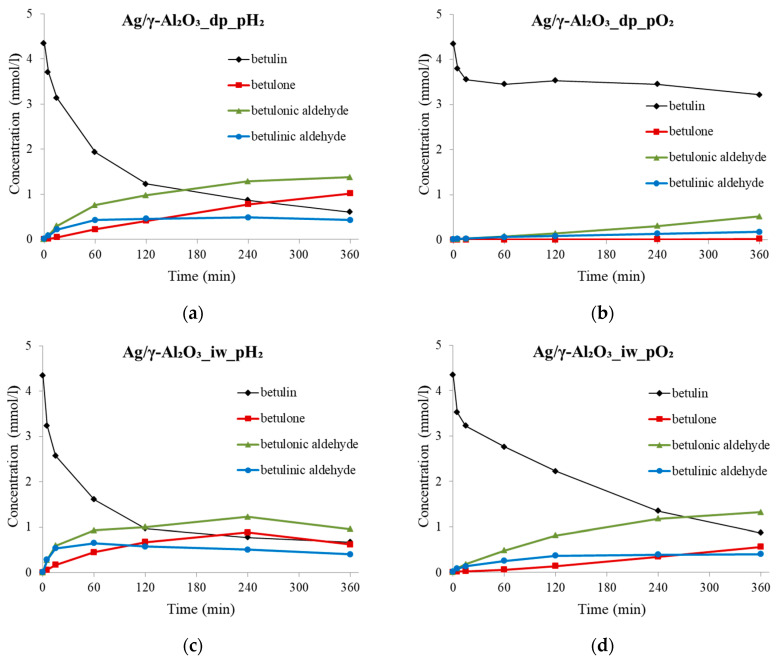

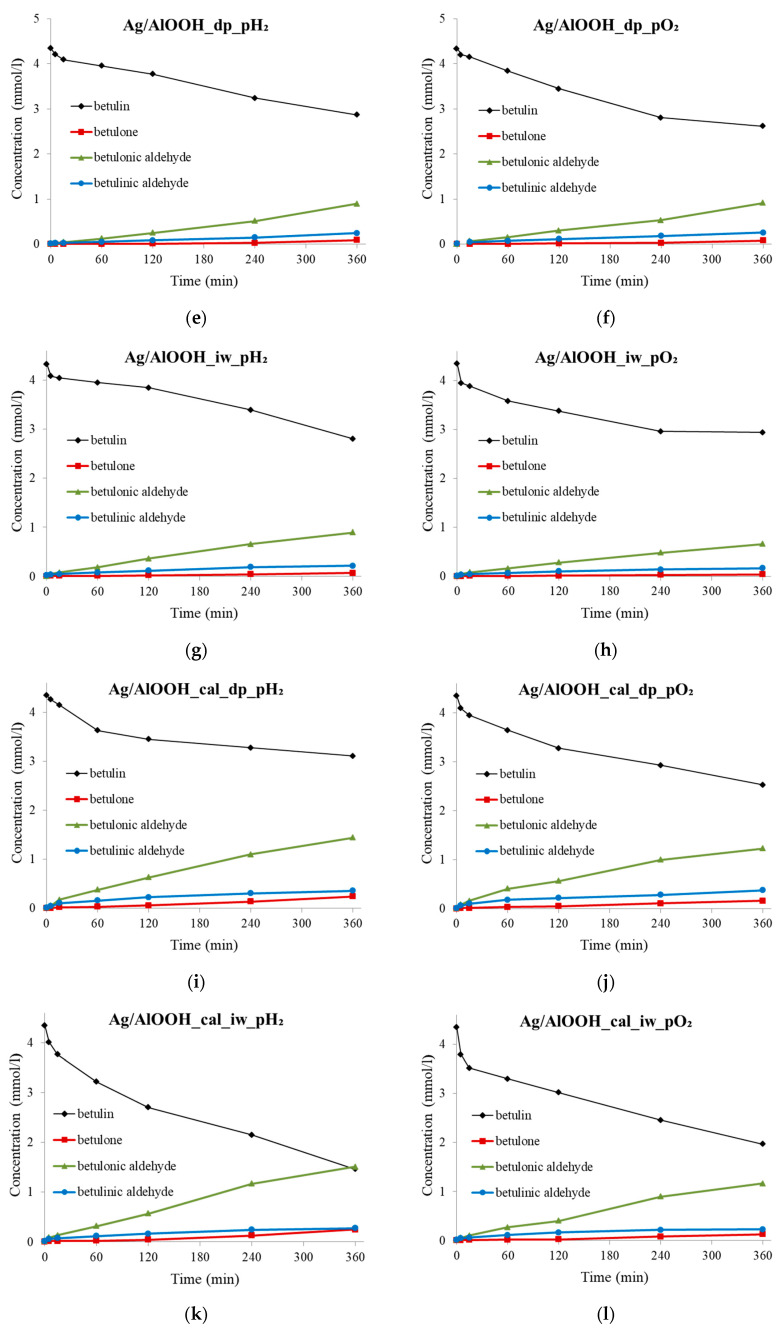
Kinetics of betulin oxidation over different silver catalysts, data are presented taking into account the mass balance closure: (**a**) Ag/γ-Al_2_O_3__dp_pH_2_; (**b**) Ag/γ-Al_2_O_3__dp_pO_2_; (**c**) Ag/γ-Al_2_O_3__iw_pH_2_; (**d**) Ag/γ-Al_2_O_3__iw_pO_2_; (**e**) Ag/AlOOH_dp_pH_2_; (**f**) Ag/AlOOH_dp_pO_2_; (**g**) Ag/AlOOH_iw_pH_2_; (**h**) Ag/AlOOH_iw_pO_2_; (**i**) Ag/AlOOH_cal_dp_pH_2_; (**j**) Ag/AlOOH_cal_dp_pO_2_; (**k**) Ag/AlOOH_cal_iw_pH_2_; (**l**) Ag/AlOOH_cal_iw_pO_2_; conditions: 140 °C, synthetic air (50 mL/min) as oxidant, solvent-mesitylene, initial betulin concentration: 4.5 mmol/L, 0.2 g catalyst.

The catalytic behavior of the supported silver catalysts was studied in the liquid-phase oxidation of betulin with synthetic air at 140 °C and mesitylene as a solvent (Table 3, Figure 3). To assess the effect of the nature of the support, the preparation method, and the pretreatment atmosphere, silver was deposited on various alumina materials (γ-alumina and boehmite) using the deposition-precipitation with NaOH (DP with NaOH) and incipient wetness impregnation (IWI) methods followed by oxidative (O_2_) or reductive (H_2_) treatment at 300 °C.

The studied Ag/alumina materials can be divided into three main groups according to their activity in betulin oxidation. The most active catalysts were found to be supported on γ-Al_2_O_3_ (Entries 1,3,4, Table 3). An exception was Ag/γ-Al_2_O_3__dp_pO_2_, which was characterized by the lowest betulin conversion (19%) and the total product yield (17%) among all studied catalysts (Entry 2, Table 3). At the same time, the material prepared by the same method and on the same support, but pretreated under H_2_, turned out to be the most active among all studied Ag/alumina materials (Entry 1, Table 3). For this catalyst conversion of betulin and the total yield of products in 6 h were 83% and 67%, respectively. The pretreatment atmosphere did not significantly affect betulin conversion and the total product yields, when materials were prepared by IWI (Entries 3 and 4, Table 3). At the same time, these values turned out to be 10–12% lower for betulin conversion and by 22–31% for the total product yield compared to Ag/γ-Al_2_O_3__dp_pH_2_ prepared by DP with NaOH (Entries 1,3 and 4, Table 3).

**Scheme 1:**
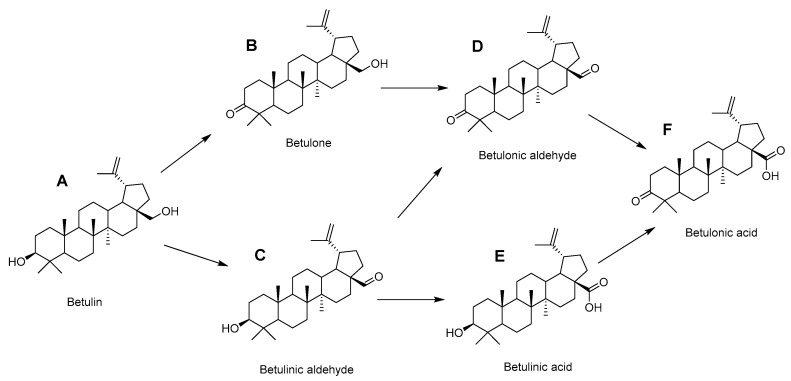
Reaction scheme for betulin oxidation. Reproduced with permission from Ref. [44]. Copyright 2019 the Royal Society of Chemistry. A—betulin, B—betulone, C—betulinic aldehyde, D—betulonic aldehyde, E—betulinic acid, F—betulonic acid.

Selectivity for the main reaction products (betulone (B), betulonic (D) and betulinic (C) aldehydes (Scheme 1), for this group of catalysts, practically did not depend on the preparation method and pretreatment atmosphere. The main reaction products were betulonic (D) and betulinic (C) aldehydes (Entries 1–4, Table 3, Scheme 1), with the former compound (D) being slightly predominant in relation to the latter one (C).

Analyzing the kinetic plots (Figure 3a,d), it can be concluded that the concentration of betulinic aldehyde (C) decreased with the reaction time. This indicates its further oxidation towards betulonic aldehyde (D), as its concentration was continuously increasing. An exception was Ag/γ-Al_2_O_3__iw_pH_2_ (Figure 3c), for which a decrease in the concentrations of all products was observed for 4–6 h. At the same time, this material displayed the worst mass balance closure (GCLPA) among all studied materials. On the whole, the mass balance closure was worse for materials prepared by IWI method (Entries 3,4, Table 3). At the same time, this value was higher for materials pretreated in O_2_ (Entries 2,4, Table 3) as compared to samples after H_2_ (Entries 1,3, Table 3). As discussed in the previous studies [44,45], the observed discrepancy between the betulin conversion values and the total yield of the reaction products is related to the side reactions, occurred on the catalyst surface with the participation of strong acid sites of the support. This issue will be discussed in more details below.

Next, in terms of activity for oxidation of betulin, were materials where AlOOH_cal was used as a support obtained by boehmite calcination at 600 °C for 6 h, giving eventually γ-alumina (Figure S1, Table 1). The betulin conversion and the total yield of the products were higher by 9–14% and 4–6%, respectively, for materials pretreated under H_2_ (Entries 9 and 11, Table 3) compared to O_2_ (Entries 10 and 12, Table 3). At the same time, betulin conversion and the total yield of the reaction products were higher for materials prepared by IWI (Entries 11 and 12, Table 3), while the mass balance closure for these catalysts was 12–18% lower than for materials prepared by DP with NaOH (Entries 9 and 10, Table 3), giving almost the same total product yields in the end.

The pretreatment atmosphere and preparation method did not significantly affect the distribution of the reaction products. The main reaction product was betulonic aldehyde (D) with an average selectivity of 70%, followed by betulinic aldehyde (C) with ca. 20%. It should be noted that in the interval of 4–6 h, there was practically no increase in the concentration of betulinic aldehyde (C), pointing out on its potential further transformations into betulonic aldehyde (D) (Scheme 1, Figure 3j–l).

Materials where boehmite (AlOOH) was used as a support displayed the lowest activity for betulin oxidation (Entries 5–8, Table 3). The maximum conversion was 24–33% after 6 h, where betulonic aldehyde (D) was the main product, with selectivity exceeding 70%. The second largest reaction product was betulinic aldehyde (C) with a selectivity varying from 18% to 20%. The mass balance closure also changed insignificantly (85–91%) and was slightly dependent on the preparation method and more on the pretreatment atmosphere. Higher values of GCLPA were detected for H_2_-treated catalysts (Entries 5 and 7, Table 3). At the same time, the total product yield for this group was 29% on average (Entries 5–7, Table 3), while a lower value (21%) was found for Ag/AlOOH_iw_pO_2_, which was also the least active among the materials in this series (24% of conversion) along with the worst mass balance closure (85%) (Entry 8, Table 3).

In general, with respect to the mass balance closure, several observed regularities can be distinguished: (a) GCLPA increases in the series Ag/γ-Al_2_O_3_ ˂ Ag/AlOOH ˂ Ag/AlOOH; (b) in each group, GCLPA is higher for the materials prepared by DP with NaOH; (c) for g/γ-Al_2_O_3_ and Ag/AlOOH_cal the mass balance closure is higher for O_2_-treated samples, while for Ag/AlOOH materials these values are higher after pretreatment in H_2_.

The observed similarities and differences with respect to the mass balance closure among the studied materials are due to the support nature, i.e., γ- alumina or boehmite, and, accordingly, their acid-base properties. Such properties of the AlOOH surface were studied in several papers [74,75,76]. Acid centers of the Lewis (LAC) and Brønsted (BAC) types with a medium and weak strength, as well as the basic Brønsted centers (BBC) were found on the surface of boehmite. There was an increase in the strength of LAC and their amount after the phase transition of boehmite to γ-alumina, which is associated with a partial decrease in the coordination of aluminum cations and compaction of the crystal lattice [77,78]. The surface of alumina oxide is covered with hydroxyl groups under ambient conditions, while such groups are removed by dehydroxylation during heating. This leads to the formation of coordination-unsaturated ions of aluminum and oxygen, i.e., acidic and basic Lewis centers (LAC and LBC), respectively. In addition, six, seven or even a larger number of absorption bands of OH-groups were found after dehydroxylation in the IR spectra of alumina. Such splitting is associated with different nature of the aluminum cations, the hydroxyl group (BAC and BBC) and their amounts.

Thus, based on results of this work and previous studies [44,45], an assumption can be put forward that the observed discrepancy between conversion and the total product yield is related with the side reactions, occurring on strong Lewis acid sites on the catalyst. Possible side reaction products include ethers and esters, which were not detected by GC due to their high molecular weight. In addition, the initial betulin contains up to 5 wt.% betulinic acid, and its concentration decreased during betulin oxidation.

To confirm the occurrence of the esterification reaction, an experiment was carried out using the equimolar ratio of betulin to betulinic acid and γ-Al_2_O_3_ as a catalyst in a nitrogen atmosphere at 140 °C. During the experiment, a decrease in the concentration of both betulin and betulinic acid was observed. However, it is worth noting that formation of ethers and esters can be catalyzed by both LAC and BAC.

At the same time, an additional argument in favor of strong alumina LAC involvement in side reactions is the difference in the mass balance closure for materials prepared by DP with NaOH and IWI methods (Table 3). The acidic properties of alumina surfaces significantly changed upon introduction of modifiers [79,80,81]. Thus, for example, alkali metals poisoned strong acid sites, while fluorination or chlorination enhanced acidity. According to [80] impregnation of γ-Al_2_O_3_ with NaOH or Ca(NO_3_)_2_ led to a decrease in the Lewis acid centers with the strongest centers disappearing first.

In the case of DP with NaOH, the weakening or disappearance of LAC can be related to the formation of sodium nitrate during precipitation, explaining a better mass balance closure for all materials prepared by this method (Entries 1,2,5,6,9,10, Table 3), compared to materials prepared by IWI (Entries 3,4,7,8,11,12, Table 3).

Separately, it should be noted that the appearance of betulinic (E) and betulonic (F) acids in the reaction products, during the betulin oxidation catalyzed by Ag/alumina materials, is practically not observed (Table 3). At the same time, as shown above, betulinic acid (E) is consumed in the esterification reaction catalyzed by the LAC of the support. The following regularity can be distinguished with regard to selectivity for betulinic (C) and betulonic (D) aldehydes: the highest selectivity for betulinic aldehyde (C) is observed for materials where γ-alumina was used as a support (up to 40%), followed by Ag/AlOOH_cal materials, while Ag/AlOOH are the least selective with respect to betulinic aldehyde (C); in the opposite direction, the selectivity to betulonic aldehyde (D) decreases (Table 3). Thus, it can be assumed that the nature of the support also determines the direction of the betulin oxidation (Scheme 1).

In the analysis of the TOF (Table 3) dependence on the average cluster size, it is necessary to take into account the influence of several factors, such as the nature of the support, the preparation method and the pretreatment atmosphere, as they affect catalytic behavior of Ag/alumina. Therefore, the studied Ag/alumina materials were divided into two groups according to their preparation method.

**Figure 4:**
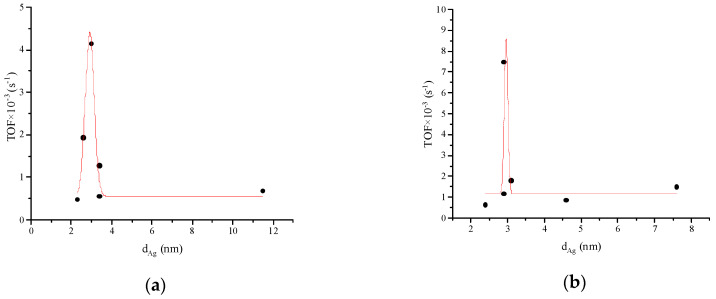
TOF dependence as a function of the mean Ag particle size: (**a**) for materials prepared by deposition–precipitation with NaOH method; (**b**) for materials prepared by incipient wetness impregnation method.

Figure 4 shows the volcano type behavior of TOF versus the Ag NP’s average size for both groups of Ag/alumina materials, i.e., those prepared by DP with NaOH (Figure 4a) and IWI (Figure 4b). As can be seen, the optimal size of Ag NPs at which the maximum TOF value can be achieved for each group of materials is in the range of 2.9–3 nm. It indicates that betulin oxidation is a structure sensitive reaction, requiring an optimal size of silver nanoparticles.

## 4. Conclusions

The present work aimed at revealing the role of the support nature, preparation and pretreatment methods in the manifestation of catalytic behavior of Ag/alumina materials for the liquid-phase oxidation of betulin with synthetic air. For this purpose, silver was deposited on the surface of various alumina materials (commercial γ-alumina, boehmite and γ-alumina obtained by calcining boehmite at 600 °C for 3 h, i.e., AlOOH_cal) using DP with NaOH and IWI methods, followed by treatment in H_2_ or O_2_. It was found that all these factors affect the catalytic behavior of the supported silver catalysts, determining not only the size and distribution of silver nanoparticles, but also the selectivity of the betulin oxidation. It was determined that the polymorphic modification of aluminum oxide predetermines the average size and distribution of Ag NPs. The smallest Ag NPs were found on the surface of materials, where γ-alumina obtained by calcining boehmite was used as a support, further materials where silver was supported on commercial γ-alumina and, finally, materials bearing silver particles on boehmite. At the same time, for each group, smaller silver particles were obtained using the DP with NaOH method. The pretreatment atmosphere also influenced the average Ag NP size and their distribution. For Ag/γ-alumina and Ag/AlOOH materials, smaller particles were obtained after H_2_-treatment, while the oxidative treatment was beneficial for Ag/AlOOH_cal. These differences were due to the different effects of the pretreatment atmosphere on particles of various sizes. However, it should be noted that the support nature had the most significant effect on the catalytic behavior of silver-containing systems.

The studied materials were divided into three groups with respect to the activity in the betulin oxidation, depending on the support. The highest betulin conversion was observed for materials with γ-alumina as a support (73–83%) and was characterized by an average size of silver particles in the range of 2.9–3.1 nm. The main reaction products for these materials were betulinic (C) and betulonic (D) aldehydes with an average selectivity of 37% and 52%, respectively. Next, in terms of activity, was Ag/AlOOH_cal materials, with an average size of Ag NPs 2.4–2.9 nm. In this case, the betulin conversion was 42–58% and the main reaction product was betulonic aldehyde (D) (71%), followed by betulinic aldehyde (C) (20%). The least active materials turned out to be materials where boehmite was used as a support (Ag/AlOOH) with the mean Ag NPs sizes varying from 3.4 to 7.6 nm. For these materials, betulin conversion was 24–33%, yielding mainly betulonic aldehyde (D) (above 70%).

Similar to recently studied gold supported on modified titania and alumina materials, for Ag/alumina there was also a discrepancy between betulin conversion and total product yield. This was attributed to an incomplete mass balance caused by side reactions, which was catalyzed by strong acid centers on the catalyst surface. Moreover, in the present study, it was found that these side reactions were catalyzed by strong LAC of the support, being therefore dependent on the support nature. In particular, Ag/γ-alumina materials were the most active among the studied catalysts; however, the mass balance closure for these materials turned out to be the worst due to the highest content of LAC. At the same time, the least active Ag/AlOOH materials, but at the same time containing the lowest amount of LAC, were characterized by the best mass balance closure. It was also found that for the same support, the mass balance closure can be increased when using deposition precipitation with NaOH for catalyst preparation, which can be explained by poisoning of strong LAC.

In addition, as with Au/alumina catalysts, the present study found that the oxidation of betulin catalyzed by Ag/alumina materials was a structure-sensitive reaction. The highest turnover frequency for betulin oxidation can be achieved for Ag NPs with the mean size of 2.9–3 nm.

## Figures and Tables

**Table 1:** Structural and textural characteristics of the studied supports and the corresponding silver catalysts, Ag content and the average Ag particle size.

Sample	Observed Phase (Framework)	S_BET_ (m^2^/g)	Pore Size (nm)	Pore Volume (cm³/g)	Ag Content (wt.%) by ICP	Ag Average Particle Size (nm) by TEM
γ-Al_2_O_3_	γ-Al_2_O_3_ (cubic)	274	11.7	1.14	-	-
AlOOH	AlO(OH) (orthorhombic)	321	5.8	0.41	-	-
AlOOH_cal	γ-Al_2_O_3_ (cubic)	248	6.6	0.53	-	-
Ag/γ-Al_2_O_3__dp_pH_2_	γ-Al_2_O_3_ (cubic) Ag (cubic)	278	11.3	1.06	2.7	3.0
Ag/γ-Al_2_O_3__dp_pO_2_	-	-	-	-	2.7	11.5
Ag/γ-Al_2_O_3__iw_pH_2_	γ-Al_2_O_3_ (cubic) Ag (cubic)	257	10.9	1.03	2.8	2.9
Ag/γ-Al_2_O_3__iw_pO_2_	-	-	-	-	2.8	3.1
Ag/AlOOH_dp_pH_2_	AlO(OH) (orthorhombic) Ag (cubic)	254	5.8	0.38	2.7	3.4
Ag/AlOOH_dp_pO_2_	-	-	-	-	2.7	3.4
Ag/AlOOH_iw_pH_2_	AlO(OH) (orthorhombic) Ag (cubic)	222	4.6	0.38	2.6	4.6
Ag/AlOOH_iw_pO_2_	-	-	-	-	2.6	7.6
Ag/AlOOH_cal_dp_pH_2_	γ-Al_2_O_3_ (cubic) Ag (cubic)	249	6.3	0.50	2.8	2.6
Ag/AlOOH_cal_dp_pO_2_	γ-Al_2_O_3_ (cubic) Al(OH)_3_ (monoclinic) Ag (cubic)	-	-	-	2.8	2.3
Ag/AlOOH_cal_iw_pH_2_	γ-Al_2_O_3_ (cubic) Ag (cubic)	232	6.5	0.61	2.7	2.9
Ag/AlOOH_cal_iw_pO_2_	γ-Al_2_O_3_ (cubic) Ag (cubic)	-	-	-	2.7	2.4

**Table 2:** Distribution of silver electronic states for the studied catalysts determined by XPS.

Sample	Ag Electronic State Relative Content, %			
	Ag^+^	Ag^0^	$Ag_{n1}^{0}$	$Ag_{n2}^{0}$
Ag/γ-Al_2_O_3__dp_pH_2_	0	81	19	0
Ag/γ-Al_2_O_3__dp_pO_2_	0	100	0	0
Ag/γ-Al_2_O_3__iw_pH_2_	7	83	10	0
Ag/γ-Al_2_O_3__iw_pO_2_	8	79	13	0
Ag/AlOOH_dp_pH_2_	0	100	0	0
Ag/AlOOH_dp_pO_2_	7	93	0	0
Ag/AlOOH_iw_pH_2_	0	100	0	0
Ag/AlOOH_iw_pO_2_	0	100	0	0
Ag/AlOOH_cal_dp_pH_2_	6	69	25	0
Ag/AlOOH_cal_dp_pO_2_	8	61	21	10
Ag/AlOOH_cal_iw_pH_2_	7	73	20	0
Ag/AlOOH_cal_iw_pO_2_	5	74	15	6

**Table 3:** Catalytic results for betulin oxidation over Ag catalysts.

Entry	Catalyst	X (%) ^1^	GCLPA (%) ^2^	TOF × 10^−3^ (s^−1^) ^3^	Selectivity (%) ^4^					∑Y_product_ (%) ^5^
					S_1_	S_2_	S_3_	S_4_	S_5_	
1	Ag/γ-Al_2_O_3__dp_pH_2_	83	77	4.13	8	53	1	38	0	67
2	Ag/γ-Al_2_O_3__dp_pO_2_	19	88	0.68	3	70	3	24	0	17
3	Ag/γ-Al_2_O_3__iw_pH_2_	75	60	7.48	12	44	2	41	tr	46
4	Ag/γ-Al_2_O_3__iw_pO_2_	73	70	1.80	9	58	1	32	0	52
5	Ag/AlOOH_dp_pH_2_	31	91	0.56	7	71	2	20	0	29
6	Ag/AlOOH_dp_pO_2_	33	86	1.27	6	72	2	20	0	29
7	Ag/AlOOH_iw_pH_2_	30	89	0.85	6	74	2	18	0	28
8	Ag/AlOOH_iw_pO_2_	24	85	1.50	5	73	3	19	0	21
9	Ag/AlOOH_cal_dp_pH_2_	51	90	1.93	7	68	2	22	tr	47
10	Ag/AlOOH_cal_dp_pO_2_	42	96	0.48	7	70	2	20	tr	41
11	Ag/AlOOH_cal_iw_pH_2_	58	78	1.17	6	72	2	19	tr	47
12	Ag/AlOOH_cal_iw_pO_2_	44	78	0.62	6	72	2	19	tr	43

^1^ X: conversion of betulin after 6 h (%); ^2^ GCLPA (the mass balance closure): the sum of reactant and product masses in GC analysis after 6 h (%); ^3^ TOF was calculated according to the equation presented in the experimental part (s^−1^); ^4^ selectivity at 30% conversion (%), with the exception of Ag/γ-Al_2_O_3__dp_pO_2_ and Ag/AlOOH_iw_pO_2_ for which selectivity is presented at maximum conversion of 19 and 24%: S_1_: to betulone; S_2_: to betulonic aldehyde; S_3_: to betulonic acid; S_4_: to betulinic aldehyde; S_5_: to betulinic acid; ^5^ ∑Y_product_: total product yield after 6 h; cal: support was calcined at 600 °C for 3 h before the synthesis of catalyst; dp: deposition–precipitation with NaOH method; iw: incipient wetness impregnation method; tr: traces.

## Data Availability

Data available upon request.

## Supplementary Materials

The following are available online at https://www.mdpi.com/2079-4991/11/2/469/s1, Figure S1: XRD patterns for studied silver catalysts and their corresponding supports.
