# Supplementary material for: Supported Silver Nanoparticles as Catalysts for Liquid-Phase Betulin Oxidation
Source: Nanomaterials (Basel). 2021 Feb 12;11(2):469. doi: 10.3390/nano11020469 (PMC7918243; doi:10.3390/nano11020469)
Supplement: Supplementary file 1 [file nanomaterials-11-00469-s001.pdf]

Supplementary Materials

Supported Silver Nanoparticles as Catalysts for Liquid-Phase Betulin Oxidation

Anna Grigoreva [1], Ekaterina Kolobova [1,*], Ekaterina Pakrieva [1], Päivi Mäki-Arvela [2], Sónia A.C. Carabineiro [3,4], Alina Gorbunova [1], Nina Bogdanchikova [5], Dmitry Yu. Murzin [2] and Alexey Pestryakov [1,*]

[1] Research School of Chemistry & Applied Biomedical Sciences, National Research Tomsk Polytechnic University, 634050 Tomsk, Russia; bar0710@mail.ru (A.G.); epakrieva@mail.ru (E.P.); aag84@tpu.ru (A.G.)

[2] Johan Gadolin Process Chemistry Centre, Åbo Akademi University, 20500 Turku/Åbo, Finland; pmakiarv@abo.fi (P.M.-A.); dmurzin@abo.fi (D.Y.M.)

[3] Centro de Química Estrutural, Instituto Superior Técnico, Universidade de Lisboa, 1049-001 Lisboa, Portugal; sonia.carabineiro@fct.unl.pt

[4] LAQV-REQUIMTE, Department of Chemistry, NOVA School of Science and Technology, Universidade NOVA de Lisboa, 2829-516 Caparica, Portugal

[5] Centro de Nanociencias y Nanotecnología, Universidad Nacional Autónoma de México, 22800 Ensenada, Mexico; nina@cnyn.unam.mx

* Correspondence: ekaterina_kolobova@mail.ru (E.K.); pestryakov2005@yandex.ru (A.P.)

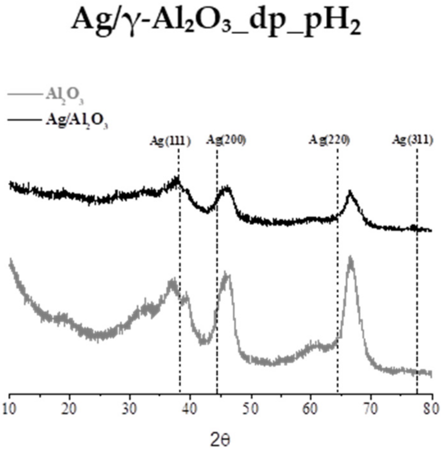

a)

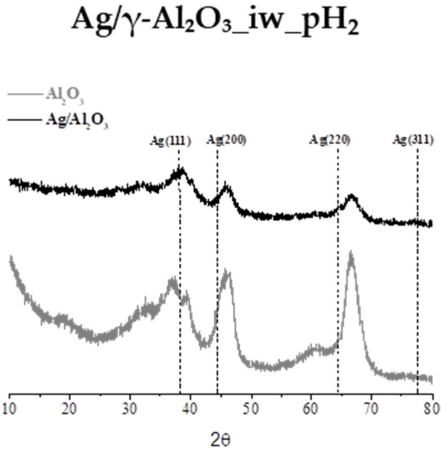

b)

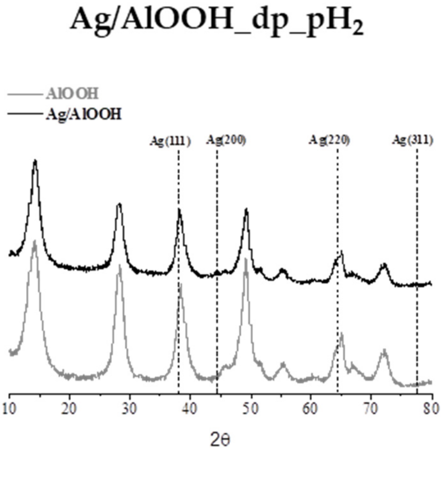

c)

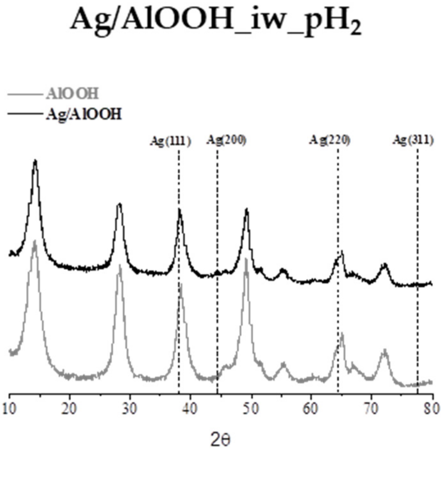

d)

Ag/AlOOH_cal_dp_as

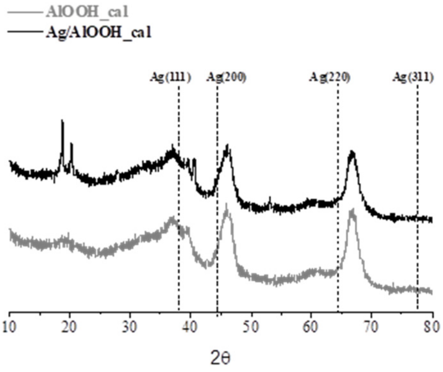

e)

Ag/AlOOH_cal_dp_pH$_2$

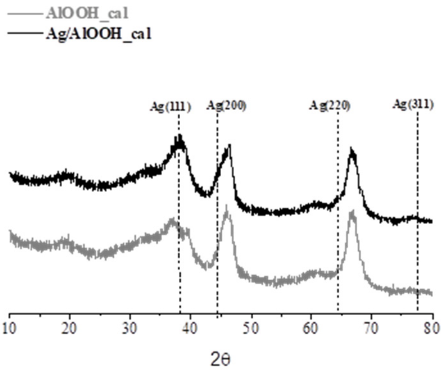

f)

Ag/AlOOH_cal_dp_pO$_2$

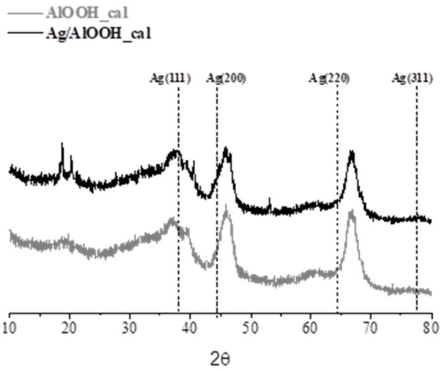

g)

Ag/AlOOH_cal_iw_as

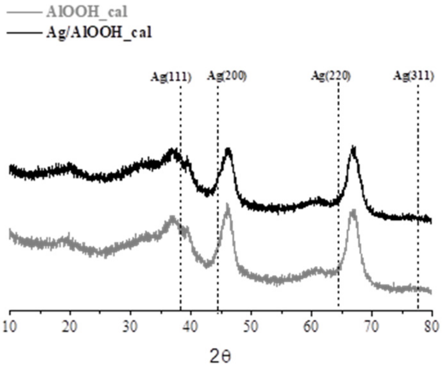

h)

Ag/AlOOH_cal_iw_pH$_2$

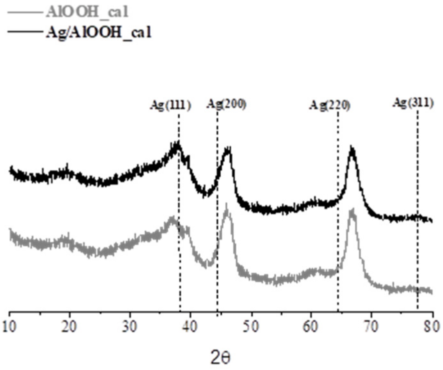

i)

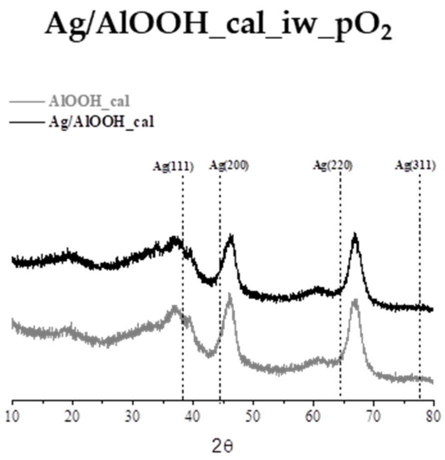

j)

Figure S1. XRD patterns for studied silver catalysts and their corresponding supports: **a)** γ-Al_2O_3 and Ag/γ-Al_2O_3_dp_pH_2; **b)** γ-Al_2O_3 and Ag/γ-Al_2O_3_iw_pH_2; **c)** AlOOH and Ag/AlOOH_dp_pH_2; **d)** AlOOH and Ag/AlOOH_iw_pH_2; **e)** AlOOH_cal and Ag/AlOOH_cal_dp_as; **f)** AlOOH_cal and Ag/AlOOH_cal_dp_pH_2; **g)** AlOOH_cal and Ag/AlOOH_cal_dp_pO_2; **h)** AlOOH_cal and Ag/AlOOH_cal_iw_as; **i)** AlOOH_cal and Ag/AlOOH_cal_iw_pH_2; **j)** AlOOH_cal and Ag/AlOOH_cal_iw_pO_2; dp—deposition–precipitation with NaOH method; iw—incipient wetness impregnation method; as—as-prepared sample; pH_2—pretreated in H_2; pO_2—pretreated in O_2.
